# Alfuzosin-Induced Autoimmune Hepatitis: A Rare Case Report

**DOI:** 10.5152/tjg.2025.25161

**Published:** 2025-06-23

**Authors:** Mustafa Ergin, Kenan Moral, Ali Karataş, Murat Kekilli

**Affiliations:** 1 Department of Gastroenterology, Aksaray University Faculty of Medicine, Aksaray Training and Research Hospital, Aksaray, Türkiye; 2 Department of Gastroenterology, Gazi University Faculty of Medicine, Ankara, Türkiye

Dear Editor,

Drug-induced liver injury (DILI) is a well-documented phenomenon, with most cases resolving upon drug discontinuation. However, some drugs can induce autoimmune-like hepatitis, termed drug-induced autoimmune hepatitis (DIAIH), which requires immunosuppressive treatment. Drug-induced autoimmune hepatitis (DIAIH) shares many clinical and histological features with idiopathic autoimmune hepatitis, while its main difference is the absence of relapse after drug discontinuation and/or steroid treatment.^1^ While DIAIH is classically associated with drugs such as minocycline, nitrofurantoin, methyldopa, and hydralazine,^2^ a rare case of alfuzosin-induced DIAIH is reported, marking the fifth reported instance of alfuzosin-associated hepatotoxicity and the first to demonstrate an autoimmune component via biopsy.

A 64-year-old male with benign prostatic hyperplasia (BPH) was prescribed alfuzosin (10 mg/day) and had been using it for 5 months without apparent adverse effects. Verbal and written informed consent was obtained from the patient. He presented with jaundice and fatigue, with laboratory results indicating severe hepatic dysfunction (aspartate aminotransferase (AST): 1139 U/L, alanine aminotransferase (ALT): 1097 U/L, alkaline phosphatase (ALP): 130, gamma glutamyl transferase (GGT): 43, total bilirubin: 9.27 mg/dL, direct/indirect bilirubin: 4.96/4.31 mg/dL, IgG: 12.14 g/L, IgM: 1.09 g/L, lactate dehydrogenase (LDH):347 U/L, creatine kinase (CK): 98 U/L, international normalized ratio (INR): 1.57). Viral hepatitis markers (HAV, HBV, HCV, EBV, CMV) were negative. Autoimmune markers (ANA, ASMA, anti-LKM, AMA) were also negative. IgG4 value was within normal range. Imaging, including magnetic resonance cholangiopancreatography (MRCP), was normal, ruling out biliary obstruction. The patient did not smoke or drink alcohol and had no history of any additional medication, herbal medicine, herbal use, mushroom consumption, or analgesic or antibiotic use.

Given the R-value of 20 (suggesting hepatocellular injury), alfuzosin was discontinued, and the patient was monitored. Despite the cessation of the drug, bilirubin levels continued to rise, prompting a liver biopsy, which showed mixed portal inflammation with plasma cells and eosinophils, suggesting DIAIH. Methylprednisolone (60 mg/day) was initiated, resulting in rapid clinical improvement, with bilirubin levels normalizing within 3 months. The steroid was tapered over 3 months, and at a 9-month follow-up, the patient remained asymptomatic with normal liver function.

Alfuzosin, a selective α-adrenergic antagonist, is extensively metabolized by the liver.^3^ Hepatotoxicity from alfuzosin is exceptionally rare, with only 4 previous cases described in the literature. In 2000, Zabala et al^4^ first reported a case of mixed-type acute liver injury from alfuzosin, which resolved after drug cessation. In 2004, Yolcu et al^5^ described hepatocellular liver injury, also resolving within 6 months post discontinuation. The most detailed case, from Korea in 2007, included a biopsy confirming cholestasis with fibrosis and necrosis.^6^ In 2015, in the case reported by Çiçek et al,^7^ liver injury detected in a patient who presented with jaundice returned to normal within days after discontinuation of the drug. However, none of these cases demonstrated autoimmune-like histopathology.

This case is unique as it is the first to report alfuzosin triggering DIAIH, confirmed via biopsy findings of plasma cell infiltration. The exact mechanism by which alfuzosin induces autoimmune hepatitis remains unclear, but drug metabolism by CYP450 enzymes may generate reactive metabolites, triggering immune activation in genetically predisposed individuals.^8^

Given the rising use of α-blockers for BPH, clinicians should be aware of this rare but potentially severe adverse effect. Regular monitoring of liver enzymes is advisable, particularly in patients who develop unexplained fatigue or jaundice.

This case highlights alfuzosin-induced DIAIH as a distinct hepatotoxic phenotype requiring steroid therapy. This emphasizes the need for early recognition, biopsy confirmation, and appropriate immunosuppressive treatment in similar cases.

## Data Availability

The data that support the findings of this study are available on request from the corresponding author.
